# ASSOCIATION BETWEEN UPPER EXTREMITY FUNCTION AND ADVERSE OUTCOMES IN OLDER ADULTS WITH COPD

**DOI:** 10.1093/geroni/igac059.2316

**Published:** 2022-12-20

**Authors:** Mehran Asghari, Martha Ruiz, Miguel Peña, Haley Johnson, Sairam Parthasarathy, Nima Toosizadeh

**Affiliations:** University of arizona, Tucson, Arizona, United States; University of Arizona, Tucson, Arizona, United States; university of arizona, Tucson, Arizona, United States; University of Arizona, Tucson, Arizona, United States; University of Arizona, Tuscon, Arizona, United States; University of Arizona, Tucson, Arizona, United States

## Abstract

Chronic obstructive pulmonary disease (COPD) is the third leading cause of death in the United States. COPD adverse outcomes are required to identify vulnerable patients and enhance bronchodilator therapies. Although the 6-minute walk distance test is commonly used to assess functional capacity in COPD and predict adverse outcomes, it is not feasible for older adults with mobility impairments. Previously, we demonstrated that a simple upper-extremity function (UEF) test could identify frailty and cognitive impairment. We developed two indexes to predict the physical frailty (20-s rapid arm test) and cognitive impairment (60-s normal speed dual-task arm test and counting) among older adults. UEF tests were performed by 76 eligible older adults (age=67.421±6.363). All participants were followed up for one month to record the adverse outcomes. The measured health outcomes included: in-hospital outcomes (death, complication, and excessive length of stay), and 30-day outcomes (30-day death or readmission). Based on the results, 54% and 30% of patients had in-hospital and longitudinal adverse outcomes, respectively. The frailty index was significantly associated with all measured outcomes (p< 0.018). However, cognitive score showed significant association only with 30-day longitudinal outcomes (p< 0.021), but not for in-hospital outcomes (p>0.05). Among all the associations the highest effect size was observed between frailty score and longitudinal outcomes (p< 0.007; effect size=0.94). The results of this study suggest that a 20-s UEF test is a practical quick measure for predicting adverse in-hospital and 30-day outcomes among COPD patients.

